# Supplementary material for: The invariances of power law size distributions
Source: F1000Res. 2016 Nov 28;5:2074. Originally published 2016 Aug 25. [Version 3] doi: 10.12688/f1000research.9452.3 (PMC5115223; doi:10.12688/f1000research.9452.3)
Supplement: Peer review history

# Peer review: Referee response for version 2

**DOI:** 10.5256/f1000research.10817.r17664

**Published:** 2016-11-16

**Authors:** Scott E. Page

**Affiliations:** 1 Department of Political Science, Center for the Study of Complex Systems, University of Michigan, Ann Arbor, MI, USA

The revised version is substantially clearer. The clarification between generic and particular on the tree model and the discussion of the log normal distribution (in my opinion) improve the paper. It's an EXCELLENT contribution.

 I now see how my previous critique was a bit of a misunderstanding on my part. The normal and log normal are really the same pattern (as stated in the appendix). Once you see that they are the same, one of the main ideas becomes much clearer.

 I have **one suggestion**. I think people will benefit by being pointed to the appendix early as the rest of the paper is more easily understood (at least by me) by first reading the description of normal, log normal, and power law because we get the functional forms. I would suggest one **modification.**

**Change the sentence:**

 "The normal distribution calls to mind the great regularity in pattern that arises solely from the aggregation of an underlying stochastic process."

**to the following**

*The familiar regular pattern exhibited by the normal distribution (or equivalently the  log normal distribution - **see appendix**) arises solely from the aggregation of an underlying stochastic process or processes.*

 Not a deal breaker for me if they don't add this but it would be a loss if they did not point to the appendix with a teaser. I also think that by saying "normal and log normal" are the same pattern, the reader will suddenly get it!!!!

I have read this submission. I believe that I have an appropriate level of expertise to confirm that it is of an acceptable scientific standard.

# Response

**Published:** 2016-11-17

**Authors:** Steven Frank

**Affiliations:** Department of Ecology & Evolutionary Biology, University of California, Irvine, USA

I have revised the article to make the suggested change, noting an equivalence of the normal and lognormal distributions and pointing to the extended discussion of the lognormal distribution in the Appendix. I am grateful to Scott Page for pushing on this issue, which has significantly enhanced the scope and reduced the chance of misunderstanding.

# Peer review: Referee response for version 1

**DOI:** 10.5256/f1000research.10181.r17202

**Published:** 2016-10-26

**Authors:** Scott E. Page

**Affiliations:** 1 Department of Political Science, Center for the Study of Complex Systems, University of Michigan, Ann Arbor, MI, USA

This is a provocative paper that should have high impact. It makes both a scholarly and educational contribution. I expect it to be widely cited and taught. When indexed, it will appear on the syllabus of a graduate class that I teach.

 That said, I have one **major **quibble with the paper. First some background.

 The standard way to teach distributions goes as follows:

1. **Normal Distributions** arise from adding or averaging variation (as nicely explained here)
2. **Log Normal Distributions **arise from multiplying shocks
3. **Power Law Distribution **have multiple causes: self organized criticality, preferential attachment, random walk return times, etc.

 

 In this paper, Frank argues that we can connect some power law and log normal distributions to normal distributions by using different unit of analysis.

 Let’s take the standard story of why tree sizes have a log normal distribution. Trees grow by random rates each year. If rates of growth are proportional, then a tree of size S that has growth rates r(t) will be size

 S[1_r(1)] [1_r(2)] [1_r(3)] [1_r(4)].. [1_r(10)]

 In 10 years. If I take the logarithm of that size, it will be additive in the shocks, and thus normally distributed.

 Frank makes an alternative argument, that there is a natural metric for size, T = d ^2^ and that this when transformed produces a normal distribution. If d is normally distributed, then d ^2^ will be log normally distributed. He then makes a similar argument for enzyme reactions.

 (The tree model is more complicated as he includes a linear term and a log term but this captures the main idea).

 My quibble with the paper has to do with the difference between a **log normal distribution **or ( **exponential**) and **a power law distribution**.  

 Power law:  y ~ x ^-a^

 Exponential: y ~e ^x^

 If you plot a log normal distribution on a log log plot, you get the sort of curvature that Frank shows in many of his graphs. What he is calling power laws would be characterized by many as log normal or exponential.

 I realize that his more general point is **not ** that the tree size is either log normal or a power law. However, the paper would be much stronger and much clearer if he would make the following changes.

1. Clarify the difference between power law and log normal (including mention of the curve on the log log plot)
2. Explain that many of the “long tails” in biology such as tree size have “long tails” that can be explained using his method that separates the generic causes of the distribution from the particular. He might even separate out the generic and particular in the tree growth so we see why he cannot just use the log normal distribution.
3. Ideally, he would also show how his approach can produce a true power law.

**I would be remiss to not add that the paper is a model of clarity of exposition and argumentation.**

I have read this submission. I believe that I have an appropriate level of expertise to confirm that it is of an acceptable scientific standard, however I have significant reservations, as outlined above.

# Response

**Published:** 2016-10-30

**Authors:** Steven Frank

**Affiliations:** Department of Ecology & Evolutionary Biology, University of California, Irvine, USA

I appreciate Scott Page’s generous summary and helpful comments. With regard to his specific suggestions:

 I agree with Page’s main point that I should relate my approach to the widely used lognormal distribution and the common power law expressions. To address those issues, I have added an Appendix to the revised version. I show how the lognormal distribution arises as an approximate description of growth and size whenever one can reasonably consider the distribution of growth rates as approximately normally distributed. Because total growth can be thought of as the sum of sequential growth increments, total growth may often be approximately normally distributed as a consequence of the various central limit theorem type arguments. However, sums of random variables are often not normally distributed, so one must be cautious about the generality of lognormal distributions for size.

 In my original article, I did not mention the lognormal distribution. I find it useful to distinguish practical applications from approaches aimed at broad conceptual understanding of the relations between different distributions. In my opinion, the lognormal distribution provides a useful practical tool, but has some limitations with regard to the conceptual aims of my article. I discuss these points in a newly added section of the Appendix.

 The new Appendix also includes a section that shows how my approach leads to a true power law distribution.

 Finally, Page discussed how one might separate the generic factors that shape probability distributions from the specific factors that influence particular observable patterns. I agree that separating the generic from the particular is a key aspect of predicting and explaining patterns. In my article, the section on “Deductive: tree size example” attempts to show how one might evaluate particular generative processes of tree growth and size within my broader framework of the invariances that shape the generic form of probability distributions. I say at the end of that section: “I presented this derivation to encourage future study. The proper way to relate general growth processes to invariant probability patterns remains an open problem.” At present, I do not have anything sufficiently compelling to add, although I certainly agree that this is a key issue. A related article of mine, “Invariant death”, emphasizes the duality of the generic and particular aspects of pattern, and adds some analysis on this topic (see https://f1000research.com/articles/5-2076/v1).

# Peer review: Referee response for version 1

**DOI:** 10.5256/f1000research.10181.r15871

**Published:** 2016-10-25

**Authors:** Neil McRoberts

**Affiliations:** 1 Department of Plant Pathology, University of California, Davis, Davis, CA, USA

This paper continues Prof Frank’s investigation of the connections between observation and probability patterns in the natural sciences, in this case dealing specifically with power law size distributions. The paper is written in a tutorial style which is probably appropriate given the that the material covered is not standard reading in the life sciences. For those who are completely unfamiliar with the general subject area, I strongly advise reading reference [3] from the list in the current paper before or in conjunction with it.

 Students in the life sciences often have a difficult relationship with concepts of probability and statistics. It should not be that way, of course, since biological systems are inherently stochastic, but it seems that as biologists we often approach the existence of variance in our observations as a problem to be got round, rather than as the very stuff of biology which it is our job to explain. Worse yet, many of us encounter the idea of “transformation” to make data more normal as an entirely ad hoc, opaque, process that seems to rely on rules of thumb learned by rote, with no explanation as to why a particular transformation would be appropriate in some situations and not others. Frank’s paper offers an altogether more satisfying perspective on the subject of transformation. In addition to a wealth of other insights, this paper lays out a well-grounded theoretical basis for understanding which transformations to seek if one wishes to preserve the information content of original, non-normal, observations but express it in terms of a corresponding normal distribution; the paper focuses in particular on the case of tree size data that conform to a power-law on their original scale of measurement. Of course, the paper is not intended as a tutorial on data transformation (the fact that students could learn to think of transformation in a new, richer, sense from reading this work is a by-product) but more an introduction to a different perspective on biological observation and its relationship with the probability distributions to which the observations conform.

 Three key ideas carry the paper along:

1. “A single underlying quantity captures the generic regularity in seemingly different patterns. That underlying quantity is the average distance of observations from the most common type.”
2. A natural metric will exist, as some transformation of the original scale of measurement, such that when considered in terms of the square root of the natural metric, average distance of observations from the most common type will follow a normal distribution.
3. With the transformation to the natural metric properly chosen, the information content of the original data distribution and the normal distribution for the data, when expressed in its natural metric, are the same (the invariance property).

The general form *p*( *y*) = *K*exp(- *L*( *y*, *x*)) in which *K* adjusts so that *p*( *y*) is a proper density function and *L(y*, *x*) is any desired distance measure is, in the words of Jorma Rissanen, a simple device used already by Gauss. In the present case it forms a density function for the distance between individual observations and the most common type. In other cases (such as those which Rissanen had in mind) it may measure the distance between model predictions and observed data. The simple device forms a link between the work presented by Frank and the extensive literature on coding, model selection and statistical inference. Exploring those links lies well beyond the scope of Frank’s paper, but their shared basis in information theory and the notion of how much, and what, information can be obtained from Nature and then modeled is an area of research that biologists have largely ignored.

 The paper includes a number of other points during the exposition of its central ideas. These, together with the complexity of the ideas themselves, and the fact that the notation used to lay out the numerous (but necessary) equations is subject to somewhat arbitrary-seeming substitutions mean that the paper needs close reading, in spite of the clarity of Frank’s writing. Although signposts are provided along the way (often in the form of rhetorical questions) to let us know where we’re going next, the paper would benefit from a more comprehensive section by section guide in the introduction, so that the whole journey can be seen in a single view.

 Returning to my initial point concerning the way that the process of making data “more normal” is often learned by rote, the sections on deduction of appropriate natural metrics to express data should be particularly useful from a pedagogic perspective; they show that (at least in theory) an approach based on argument from principles is possible. Those who are unfamiliar with the ideas will still probably be left with the unwelcome impression that it will take considerable experience to become proficient at recognizing approaches that are likely to work, but one of the wider lessons of Frank’s publications in this general area is that rather few approaches are likely to account for the majority of observations most of us will encounter.

I have read this submission. I believe that I have an appropriate level of expertise to confirm that it is of an acceptable scientific standard.

## References

[1] FrankSA: The common patterns of nature. *J Evol Biol*.2009;22(8) : 10.1111/j.1420-9101.2009.01775.x 1563-85 10.1111/j.1420-9101.2009.01775.x 19538344PMC2824446

# Response

**Published:** 2016-10-27

**Authors:** Steven Frank

**Affiliations:** Department of Ecology & Evolutionary Biology, University of California, Irvine, USA

Neil McRoberts’ summary provides an excellent overview of the broader subject and the place of my article within that wider context.

 McRoberts mentions the duality between the understanding of probability patterns and the complementary problems of inference. I originally came to this subject through that connection, particularly through my study of Jaynes pioneering work (see references to my earlier work in my article). However, I was not aware of several of the explicit connections mentioned by McRoberts, which I appreciate learning about from his review.

 McRoberts suggests that I provide “a more comprehensive section by section guide in the introduction.” I often provide such a guide in my longer articles. In this case, I had that kind of guide in my early drafts. However, the technical underlying nature of the work made the overview into what seemed like more of an obstacle than an invitation to the article. So I dropped it, allowing me to move the article very quickly into the example of tree size that I use throughout to help connect the underlying abstractions to real-world problems. Perhaps it would be possible to write a helpful introductory guide, but I have not yet found the right expression.

 The main difficulty with the current structure is that some readers may mistakenly focus on the tree size problem as the central message of the article. It is not. The main message is that we can understand almost all common probability patterns by a few simple underlying invariances. That understanding provides great insight into many aspects of commonly observed patterns, including patterns such as tree size.

 One advantage of the F1000Research format is that I can submit a revised version at any time. For now, I will keep the current structure, while I continue to think about how to improve the presentation. I welcome comments from readers.
